# TSSG-CNN: A Tuberculosis Semantic Segmentation-Guided Model for Detecting and Diagnosis Using the Adaptive Convolutional Neural Network

**DOI:** 10.3390/diagnostics14111174

**Published:** 2024-06-01

**Authors:** Tae Hoon Kim, Moez Krichen, Stephen Ojo, Meznah A. Alamro, Gabriel Avelino Sampedro

**Affiliations:** 1 School of Information and Electronic Engineering, Zhejiang University of Science and Technology, No. 318, Hangzhou 310023, China; 2 ReDCAD Laboratory, University of Sfax, Sfax 3038, Tunisia; 3 Department of Electrical and Computer Engineering, College of Engineering, Anderson University, Anderson, SC 29621, USA; sojo@andersonuniversity.edu; 4 Department of Information Technology, College of Computer & Information Science, Princess Nourah Bint Abdul Rahman University, Riyadh 11564, Saudi Arabia; meaalamro@pnu.edu.sa; 5 Faculty of Information and Communication Studies, University of the Philippines Open University, Los Baños 4031, Philippines; garsampedro@ieee.org; 6 Gokongwei College of Engineering, De La Salle University, 2401 Taft Ave., Malate, Manila 1004, Philippines

**Keywords:** tuberculosis, convolutional neural network, segmentation model, healthcare, deep learning

## Abstract

Tuberculosis (TB) is an infectious disease caused by Mycobacterium. It primarily impacts the lungs but can also endanger other organs, such as the renal system, spine, and brain. When an infected individual sneezes, coughs, or speaks, the virus can spread through the air, which contributes to its high contagiousness. The goal is to enhance detection recognition with an X-ray image dataset. This paper proposed a novel approach, named the Tuberculosis Segmentation-Guided Diagnosis Model (TSSG-CNN) for Detecting Tuberculosis, using a combined semantic segmentation and adaptive convolutional neural network (CNN) architecture. The proposed approach is distinguished from most of the previously proposed approaches in that it uses the combination of a deep learning segmentation model with a follow-up classification model based on CNN layers to segment chest X-ray images more precisely as well as to improve the diagnosis of TB. It contrasts with other approaches like ILCM, which is optimized for sequential learning, and explainable AI approaches, which focus on explanations. Moreover, our model is beneficial for the simplified procedure of feature optimization from the perspectives of approach using the Mayfly Algorithm (MA). Other models, including simple CNN, Batch Normalized CNN (BN-CNN), and Dense CNN (DCNN), are also evaluated on this dataset to evaluate the effectiveness of the proposed approach. The performance of the TSSG-CNN model outperformed all the models with an impressive accuracy of 98.75% and an F1 score of 98.70%. The evaluation findings demonstrate how well the deep learning segmentation model works and the potential for further research. The results suggest that this is the most accurate strategy and highlight the potential of the TSSG-CNN Model as a useful technique for precise and early diagnosis of TB.

## 1. Introduction

Mycobacterium tuberculosis is the bacterium that causes tuberculosis (TB) [1]. Although the renal system, spine, and brain may also be impacted, the lungs are the primary organs. Because it spreads through the air when an infected person coughs, sneezes, or talks, TB is incredibly contagious, [2]. Despite its preventable and treatable nature, TB remains a major global health concern, particularly in low- and middle-income countries and among vulnerable populations, such those who are HIV/AIDS-positive [3]. TB can have a serious negative impact on both human health and national health systems. Exhaustion, fever, weight loss, and coughing are common symptoms of the condition [4]. If treatment is not administered, TB can have serious implications, such as lung damage, respiratory failure, and even death [5]. The fact that TB typically necessitates a protracted course of antibiotics and may require multiple medications to combat drug-resistant strains exacerbates treatment complexity and adherence concerns [6].

Global health concerns about TB are still significant, especially in areas where access to healthcare resources is scarce [7]. Effective TB treatment and control depend on early detection and accurate diagnosis. Chest X-rays are a commonly used diagnostic method for TB screening, since they are inexpensive and easy [8]. It can be challenging to interpret chest X-rays for the diagnosis of TB, especially in places with few resources and a lack of radiology with the necessary training [9]. Recent advancements in deep learning techniques have shown the promise for automated identification of TB from chest X-ray images [10]. This article proposes a novel approach integrating segmentation and classification models to enhance the accuracy of TB detection employing chest X-rays. Specifically, X-rays were used, as they are convenient to store, transmit, and analyze, plus they are easily accessible and are cost-effective for every patient. By combining segmentation techniques to identify areas of concern with algorithms for classification to distinguish between patients who are TB-positive and those who are TB-negative, this combination strategy aims to improve TB diagnosis sensitivity and specificity.

### 1.1. Motivation

This work is motivated by the need to improve TB diagnosis and screening, particularly in impoverished areas where access to trained medical personnel is limited. Traditional methods of TB detection that solely depend on the laborious, subjective manual analysis of chest X-rays may lead to delayed therapy initiation and inaccurate diagnosis [11]. Moreover, the majority of automatic TB detection systems now focus solely on classification algorithms, neglecting the importance of accurately locating relevant regions within chest X-rays. The objective of this research is to surmount these limitations and enhance the accuracy and effectiveness of TB detection using chest X-ray images by merging segmentation techniques with classification models. This work attempts to expand on prior research by using knowledge from the literature study of deep learning-based TB detection methods to create a more reliable and efficient strategy for TB prevention using sophisticated image analysis techniques.

### 1.2. Contributions

- We propose a novel approach to tuberculosis diagnosis from X-ray images named the Tuberculosis Segmentation-Guided Diagnosis model (TSSG-CNN) that combines semantic segmentation and the adaptive CNN model. Such an approach is extremely beneficial as compared to conventional segmentation and classification algorithms that cope with the problem independently from each other; they improve the detection of TB by directing attention to the concrete areas that are depicted on the image before the classification process.
- We contribute towards detecting TB by improving accuracy through the cutting-edge proposed segmentation model. A comparison between our model and newly emerging algorithms like Incremental Learning-based Cascaded Models and explainable AI (XAI) models proves that our model has better performance. The gains in accuracy and F1-score over previous techniques confirm the efficiency and dependability of the suggested TSSG-CNN for TB identification. The proposed model addresses the limitations of the previous approaches by providing robustness and precision.
- After evaluation, the results demonstrated that TB can be detected with an accuracy of 98.75%. The test results on unseen images validate that the introduced deep learning approach is the most effective and accurate.

### 1.3. Organization

This paper is organized into five sections. Section 1 provides a brief introduction to the topic, while Section 2 covers the literature review and the previous studies on TB detection. Section 3 and Section 4 elaborate on the proposed approach and the experimental results gained from the study. Finally, Section 5 concludes the paper with future research that can be conducted.

## 2. Literature Review

The authors in [12] presented a novel method for identifying TB in chest X-ray (CXR) photos in response to the need for faster and more accurate diagnosis techniques. The “Incremental Learning-based Cascaded Model” (ILCM), which they presented, offers two crucial features: it can identify infected areas within the CXR picture and classify TB cases. By automating these processes, the ILCM reduces the workload for medical staff and allows for quicker TB patient discovery and treatment commencement. Both a benchmark golden standard dataset and data from the local population are used to assess the model’s efficacy. The ILCM obtains an F1 score of 97.23% and a remarkable overall accuracy of 93.20% on the local data. For comparison, it obtains a remarkable 83.32% overall accuracy and 82.24% F1 score on the benchmark dataset. The calculation results show that the ILCM will produce a high accuracy and F1 score. Still, the efficient performance can be affected by the better or worse training datasets and the diversity levels. Choosing the individuals representative of subpopulations or TB variants may limit the external validity of the model.

In order to identify TB in chest X-rays, Ref. [13] presented a novel method that blends explainable Artificial Intelligence (XAI) techniques with the CNN framework. With CNNs, the model can detect differences between TB-positive and TB-negative patients in medical images, including chest X-rays. The accuracy falls between 98.7% and 99.1%. Additionally, the use of XAI makes it easier to understand CNN’s decision-making process by making it clear which features and regions of the X-rays are most important for the diagnosis of TB. The study likely includes data collection from patients who have been diagnosed with TB and a control group. The generated dataset is then used to train CNN, and XAI is included for interpretability. The reliability of the high accuracy reported is often overestimated, and the risk of overfitting is high, especially when the dataset is not diverse, either because it is experimentally ineffective or the distribution of TB-positive TB-negative cases is unbalanced. Also, the interpretability derived from the XAI might be bounded by a challenge of the CNN architecture and the interpretability applicability.

In [14], a novel approach to feature optimization utilizing the Mayfly Algorithm (MA) and Dual Deep Learning Features were presented for the detection of TB in chest X-rays. Utilizing characteristics from previously trained models VGG16 and VGG19, the method looks for intricate patterns in X-rays that are required for diagnosing TB. Next, the MA selects and refines the most important features to increase the efficacy and precision of TB categorization. The work uses a KNN classifier for TB detection and achieves remarkable accuracy rates of 97.8% using a variety of procedures, including feature extraction, data collecting, and optimization with the MA. The MA-based optimization of features in the proposed method is dataset and model-type dependent. It should also be noted that the use of a KNN classifier also poses limitations to the scalability and efficiency of the algorithm in terms of the design of a real-world application with a large number of objects.

A novel approach to early TB diagnosis through deep learning integration into an Internet of Things (IoT)-based healthcare application is presented in the paper [15]. The primary screening method, which uses chest X-ray images, acknowledges the significance of early detection in TB therapy. The process includes data preparation, potential segmentation using Adaptive Fuzzy C-means clustering, and deep learning-driven extraction of features made possible by a Deep Belief Network (DBN) for classification. To optimize performance, the paper employs the Adaptive Monarch Butterfly Optimization (AMBO) method. The results offer promising outcomes, demonstrating the superiority of the DBN-AMBO combination over competing procedures and potentially improving the reliability of TB detection, even though the exact accuracy is not defined. A few potential limiting factors in relation to the proposed IoT-based healthcare application include network connectivity, privacy and confidentiality of data handling, and availability in different settings. Also, the use of deep learning models for feature creation demands much computational time, thus making their use impractical in environments with resource constraints.

The authors in [16] used deep learning techniques in a unique pipeline for automated TB screening utilizing chest X-rays, offering a ground-breaking solution to the urgent global problem of TB detection. The study tackled the subjectivity and time limits associated with human interpretation of chest X-rays, especially in resource-limited situations, acknowledging the criticality of early identification in the fight against TB. To improve performance, the pipeline that is being suggested combines three sophisticated deep learning architectures and strategically applies methods, including picture pretreatment, augmentation, genetic algorithm optimization, and model ensembling. The system outperforms current techniques with an astounding 97.1% classification accuracy, and strong measures like Youden’s index, sensitivity, and specificity confirm its efficacy even more. The high classification accuracy is, however, dependent on the quality and resolution of input chest X-ray images from deep pipeline definition. Secondly, the pipeline is computationally complex, and thus, non-realistic for use in resource-limited environments.

The authors in [17] offered a novel deep learning model called CBAMWDnet (Convolutional Block Attention Module Wide Dense Net), designed especially to use chest X-ray (CXR) pictures for the early identification of TB. Convolutional Block Attention Module (CBAM) and Wide DenseNet (WDnet), two essential components, are combined by CBAMWDnet to improve its capacity to identify critical characteristics in CXR pictures that are necessary for TB identification. Comprehensive information extraction is ensured by the WDnet, which effectively learns characteristics through dense connections, while the CBAM prioritizes pertinent information across various regions of the CXR. It is noteworthy that CBAMWDnet outperforms other models with an astounding 98.80% accuracy rate in TB detection, and it performs admirably in terms of sensitivity, precision, specificity, and F1 score, among other evaluation metrics. The training data size and diversity of the training data also affect the training of the CBAMWDnet model, as well as the selection of the hyperparameters during training. Moreover, the time required to train models and test models further presupposes enormous computations that would be impossible to support in resource-scarce environments.

The authors in [18] addressed the shortcomings of conventional methods that frequently concentrate on individual diseases like pneumonia, COVID-19, and TB. Instead, it proposes a novel way of detecting lung disorders using chest X-rays. A deep learning model that can jointly diagnose these four lung disorders is shown in the suggested solution. The model is trained on Kaggle datasets that are made publicly available, and it performs remarkably well, diagnosing all four disease categories with an accuracy of 98.72%. It also performs well in distinguishing specific disorders, as seen by recall scores higher than 96% for each. Experiments were conducted on data that were not seen to validate the effectiveness of the model, surpassing existing methods in precision. This discovery significantly enhances lung illness diagnosis over traditional single-disease-focused approaches, offering a more efficient and probably more accurate approach. Deep learning, combined with the detection of multiple lung disorders, has a bright future, but the performance of the model may be impaired in cases of coexisting diseases or overlapping signs of different lung disorders. Second is the fact that such solutions rely on the Kaggle datasets, and there is a danger of over-fitting and potential biases towards the representativeness of the information.

The authors in [19] discussed the critical need for early diagnosis in high-burden countries, particularly in situations when access to trained radiologists is scarce. They introduced a novel computer-aided diagnostic (CAD) method for automated chest X-ray-based TB identification. The technique blends manually created characteristics that are recovered using Gabor filters—which are well-known for capturing specific textures and patterns—with deep features from pre-trained deep learning models. This combination offers a comprehensive way of tuberculosis detection, reducing the requirement for valuable personnel and perhaps enhancing efficiency as compared to manual screening approaches. Robust examination of the system using k-fold cross-validation on two publicly available datasets demonstrates its good performance. For both the Montgomery dataset and the Shenzhen dataset, the evaluation produced remarkable areas under the receiver operating characteristic curve (AUC) values of 0.97 and 0.99, respectively. The CAD method performs well on open datasets. Still, in the real clinical world, this effectiveness can be affected by data quality, patient demographics, and differences in various imaging protocols. Deep learning, combined with the detection of multiple lung disorders, has a bright future. Still, the performance of the model may be impaired in cases of coexisting diseases or overlapping signs of different lung disorders. Second is the fact that such solutions rely on the Kaggle datasets, and there is a danger of over-fitting and potential biases towards the representativeness of the information.

To address the issue of class disparity in chest X-ray datasets used to diagnose TB, this paper [20] focuses on the TBX11K dataset, which is prominent for having an uneven distribution of instances that are TB-positive and TB-negative. To mitigate the effects of this imbalance, the study looks at the effectiveness of the Synthetic Minority Over-sampling Technique (SMOTE), a method for producing synthetic data points to balance the classes. This study evaluates each model’s performance in terms of F1 score, accuracy, precision, recall, and SMOTE augmentation. It employs the Random Forest (RF) and XGBoost (XGB) models for classification. The results demonstrate that while SMOTE enhances the precision–recall trade-off, the total accuracy of both models is somewhat worse. Despite a slight decrease in accuracy, the outcomes demonstrate how effective SMOTE is in addressing disparities in classes. The RF model without SMOTE is still the best option when overall accuracy is the top priority, but the XGB model with SMOTE is recommended for TB case identification. Class disparity in datasets is indeed important; however, the performance of SMOTE augmentation might not be as expected, but it is dependent on the distribution as well as the distinctive features that characterize it. What is more, results obtained from this approach—along with any selected classification models—may not be applicable broadly to other sets of data and people. For instance, different distributions could have different modes or means; therefore, applying such a model could result in biases, especially towards underrepresented groups.

The authors in [21] explored how deep learning models can be used to improve chest X-ray quality control (QC) for TB testing in immigrants and refugees visiting the US. Traditional quality control methods used by the US Centres for Disease Control and Prevention (CDC) need to be more scalable. The proposed models demonstrate exceptional performance in identifying anomalous X-rays and anomalies linked to TB, as demonstrated by their outstanding area under the curve (AUC) of 0.97 for anomalies related to TB on internal data, following their training on a large dataset. They also forecast the frequency of aberrant X-rays with an accuracy and error margin of 2%. Performance is still good on external datasets, with AUCs that range from 0.89 to 0.99. A few considerations that affect the accuracy of deep learning algorithms in chest radiography quality assurance include image resolution, inconsistency among imaging devices, and unrelated TB artifacts or irregularities. Moreover, more exploration is needed on how well these models can be applied to various demographic patient populations and differing healthcare system contexts.

As summarized in Table 1, these findings demonstrate the immense potential of deep learning in scaled and accurate TB diagnosis, which offers significant advantages in public health screening programs for immigrant and refugee communities.

## 3. Proposed Approach

**Figure 1:**
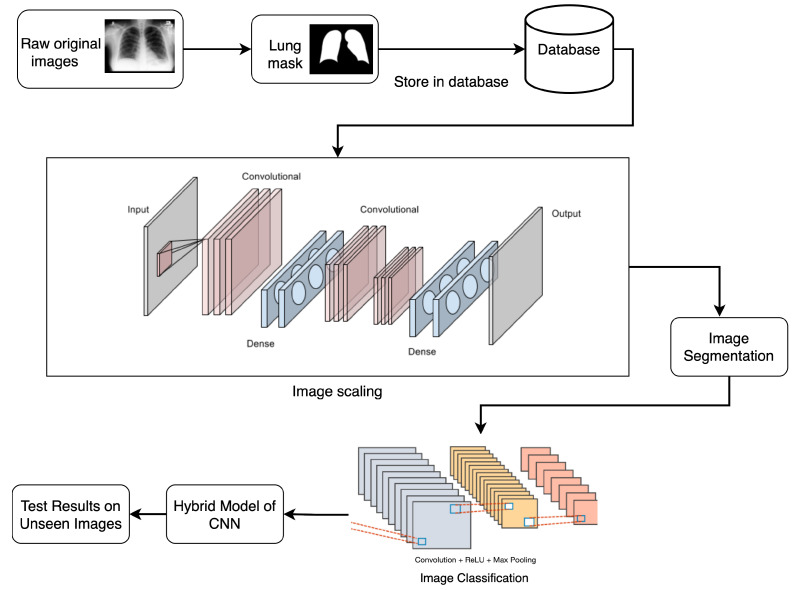
Workflow of the proposed approach.

This section outlines the steps of our proposed approach, including details on the dataset, preprocessing, and the suggested Tuberculosis Segmentation-Guided Detection Model (TSSG-CNN), which is based on a CNN-based segmentation model and classification model. Figure 1 provides a graphic depiction of the suggested strategy. Firstly, the raw X-ray images were masked to provide an image that the model could understand easily; once completed, they were stirred and scaled before giving the images for segmentation. After segmenting the images, they were classified through the CNN model, and finally, a model was trained on this dataset to predict TB.

### 3.1. Dataset and Preprocessing

**Figure 2:**
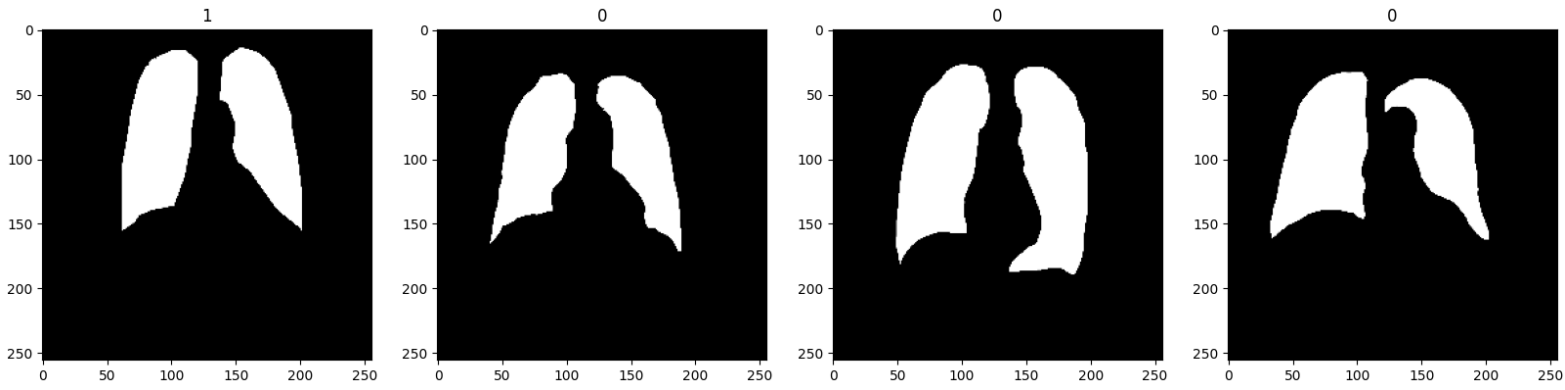
Sample of the dataset.

The Shenzen Hospital X-ray set’s manually categorized lung masks are included in the Kaggle dataset that was used to build the model [22]. The dataset consists of 566 images that were masked using lung segmentation that isolated the lung region within the X-ray images for refining the model’s focus; also, rotations and flips are two examples of data augmentation strategies that are highlighted for increasing dataset size and enhancing model generalization [23]. The masked images are a binary class dataset further used for detecting TB; masking helped to generate better results even with the small dataset with less than 1000 images. The dataset images were kept in 18 batches, out of which 12 were used for training, 5 for validation, and 1 for testing. Before giving this dataset as an input to the model, data scaling was performed to range the images between [0, 1] by dividing every image by 255, ensuring consistency for model training. Figure 2 represents the sample data that is used for training the model.

### 3.2. Tuberculosis Segmentation-Guided Detection (TSSG-CNN) Model

**Figure 3:**
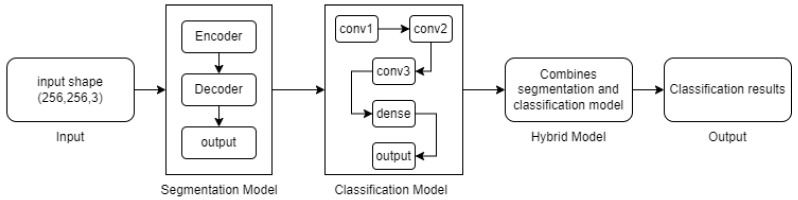
Classification flow of the proposed model.

The proposed TSSG-CNN model consists of a segmentation model and a classification model that is then combined by a hybrid model as seen in Figure 3. Algorithm 1 explains how to use TensorFlow and Keras to create and train a hybrid segmentation classification model. Convolutional layers for feature extraction and upsampling layers for segmentation mask creation make up the segmentation model, which is first defined. Next, the convolutional and dense layers that make up the classification model for image classification tasks are defined. After that, the hybrid model is created by combining the segmentation and classification models. Ultimately, training data are used to construct and train the hybrid model, while validation data are used to track performance indicators. The methodology offers an organized method for creating and honing a deep-learning model that can process input photos for both segmentation and classification purposes.
**Algorithm 1:** Creating and Training the Hybrid Segmentation-Classification Model**Require:** Input Shape = (256, 256, 3)
   1: **Define Segmentation Model**
   2: inputs ← Input(input_shape)
   3: conv1 ← Conv2D(32, (3, 3), activation=`relu’, padding=`same’)(inputs)
   4: pool1 ← MaxPooling2D(pool_size=(2, 2))(conv1)
   5: up1 ← UpSampling2D(size=(2, 2))(pool1)
   6: decoded ← Conv2D(3, (3, 3), activation=`sigmoid’, padding=`same’)(up1)
   7: segmentation_model ← Model(inputs, decoded, name=`segmentation_model’)
   8: **Define Classification Model**
   9: inputs ← Input(input_shape)
 10: conv1 ← Conv2D(16, (3, 3), activation=`relu’)(inputs)
 11: pool1 ← MaxPooling2D(pool_size=(2, 2))(conv1)
 12: conv2 ← Conv2D(32, (3, 3), activation=`relu’)(pool1)
 13: pool2 ← MaxPooling2D(pool_size=(2, 2))(conv2)
 14: conv3 ← Conv2D(64, (3, 3), activation=`relu’)(pool2)
 15: pool3 ← MaxPooling2D(pool_size=(2, 2))(conv3)
 16: flatten ← Flatten()(pool3)
 17: dense1 ← Dense(256, activation=`relu’)(flatten)
 18: output ← Dense(1, activation=`sigmoid’)(dense1)
 19: **Combine Segmentation and Classification Models**
 20: input_tensor ← Input(input_shape)
 21: segmentation_model ← create_segmentation_model()
 22: segmentation_output ← segmentation_model(input_tensor)
 23: classification_model ← create_classification_model()
 24: classification_output ← classification_model(segmentation_output)
 25: hybrid_model ← Model(inputs=input_tensor, outputs=classification_output, name=`hybrid_model’)
 26: **Compile and Train the Hybrid Model**
 27: hybrid_model.compile(optimizer=`adam’, loss=`binary_crossentropy’, metrics=[`acc’])
 28: logdir=`logs’
 29: hist ← hybrid_model.fit(train, epochs=25, validation_data=val, callbacks=[tensorboard_callback])


#### 3.2.1. Segmentation Model

The segmentation model consists of a convolutional neural network (CNN) encoder-decoder architecture that is commonly used for image segmentation where the model itself learns to assign labels to each pixel in the input image [24]. Convolutional layers are placed in the “encoder” portion of the architecture, which is followed by pooling layers. These layers extract high-level characteristics while gradually decreasing the input image’s spatial dimensions. This process is reversed by the “decoder” in the architecture, which creates an output map with the same spatial dimensions as the input image by upsampling the feature maps that are produced by the encoder. Convolutional layers work by convolving filters across the input image during the feature extraction process, locating specific patterns and characteristics that are necessary for further analysis as presented in Equation (1):

(1) $$\mathrm{convolution}=\sum_{i=1}^{N}\left(\mathrm{input}\times \mathrm{filter}+\mathrm{bias}\right)$$

Then, non-linear transformations are introduced using activation functions like ReLU, which improve the network’s ability to identify complex links in the data, as shown in Equation (2). Then, by choosing the largest value inside each pooling region, max-pooling layers minimize the spatial dimensions of the feature maps that are presented by Equation (3):

(2) ReLU(x)=max(0,x)

(3) MaxPooling(x)=max(Pooling_Region(x))

This efficiently summarizes the learned features and improves the model’s resistance to spatial fluctuations in the input data. This multi-step procedure not only makes robust feature extraction easier but also makes a big difference in how effective the model is overall for tasks like segmentation and classification. In the Upsampling and Segmentation Mask Generation stage, the decoder reverses the encoder’s downsampling procedure to recover the spatial dimensions of the original input image. Methods like interpolation and transposed convolutions are frequently employed for this. The final decoder layer uses sigmoid activation to provide pixel-wise predictions or the likelihood of particular groups or categories for each pixel. By creating segmentation masks, this procedure helps with tasks like object detection and medical image analysis by defining zones of interest within the input image.

#### 3.2.2. Classification Model

A convolutional neural network (CNN) architecture specifically designed for image classification problems is used in the classification model. This architecture makes it easier to extract hierarchical characteristics from the input images by including numerous convolutional layers, each followed by max pooling operations. Convolutional layers efficiently detect local patterns and features, while feature abstraction and computing efficiency are improved by max pooling, which decreases spatial dimensions. The learned features are then represented as vector representations by flattening the feature maps, which are subsequently run through several thick layers to carry out classification. Dense layers—also referred to as completely connected layers—allow the model to represent intricate feature interactions; Equation (4) represents the dense layer:

(4) Dense(x)=σ(W·x+b)

The output layer predicts the probability that an input image will belong to a specific class using the sigmoid activation function; the activation function is represented in Equation (5):

(5) $$\mathrm{sigmoid}\left(x\right)=\frac{1}{1+e^{-x}}$$

This produces a binary result that is commonly utilized for binary classification tasks. With this all-encompassing method, the model can efficiently classify photos into different categories according to learned features, which makes it appropriate for a range of image classification uses.

#### 3.2.3. Hybrid Model

Joint segmentation and classification tasks on input images are made possible by the Hybrid Model, which combines the features of both the segmentation and classification models. By examining the input images, the segmentation model first creates segmentation masks, or boundaries, around particular regions or objects in the image. The classification model then takes these segmentation masks as input, using the segmented regions to perform binary classification tasks. By employing this technique, the model may consider both the global context of the image and the segmented localized aspects. By integrating segmentation and classification abilities, the hybrid model can provide comprehensive insights into the presence or absence of specific features within the input images.

Additionally, the model is compiled using the binary cross-entropy loss function and Adam optimizer to make it optimal for learning segmentation and classification tasks. The accuracy assessment metric confirms that the model is effective in accurately segmenting and classifying input images. For difficult image analysis tasks, the hybrid technique offers a reliable and flexible solution, particularly when continuous segmentation and classification are required.

### 3.3. Pseudo Code for TB Detection

Pseudocode in Section 3.3 of the proposed model explains the working of the model’s input, its segmentation, its classification, and its results. The model accepts an input shape of (256 × 256 × 3) that is given to the segmentation model, where the input image is encoded using conv2D and maxpooling2D layers. Then, it is decoded using upsampling2D and conv2D layers, and it provides the segmented images that are then given to the classification model as input. The images are further classified using multiple conv2D and maxpooling2D layers; furthermore, in the classification process, one flattened layer and two dense layers are utilized to finalize the classification. Finally, a hybrid model is designed using both segmentation and classification models.

1. Define input shape for images (256x256x3).
2. Create a segmentation model:
  a. Input layer.
  b. Encoder with Conv2D and MaxPooling2D layers.
  c. Decoder with UpSampling2D and Conv2D layers.
  d. Output layer with sigmoid activation.
3. Create a classification model:
  a. Input layer.
  b. Series of Conv2D and MaxPooling2D layers.
  c. Flatten layer.
  d. Dense layers with ReLU activation.
  e. Output layer with sigmoid activation.
4. Combine models:
  a. Use segmentation model output as input to the classification model.
  b. Define the hybrid model.
5. Compile and summarize the hybrid model.

## 4. Experimental Analysis and Results

This section provides a comprehensive review of the assessment measures used to evaluate the performance of the TSSG-CNN model. Evaluation metrics reveal how well a model can recognize patterns and predict outcomes, making them crucial indicators of a model’s performance. The process of evaluating the TSSG-CNN model is a multimodal analysis that evaluates the model’s overall performance using a variety of metrics.

Accuracy is a crucial parameter for evaluating classification models, since it measures the overall reliability of predictions by calculating the ratio of correctly predicted instances to all instances in the dataset. In unbalanced datasets, when one class is predominant and more evaluations are needed, its value may be compromised. In scenarios like fraud detection or medical diagnosis, where false positives can be costly, precision estimates the proportion of true positive predictions among all positive predictions. The recall is an estimation of the model’s ability to identify real positive occurrences relative to all positive instances, and it is important for situations that prioritize catching positive events. It goes by the names true positive rate and sensitivity as well. The F1-score, which is a harmonic mean of precision and recall, balances these two measures to give a comprehensive evaluation of a model’s performance when both precision and recall are equally significant or when class distributions are uneven. When combined, these metrics offer a comprehensive understanding of a classification model’s capabilities, empowering academics and practitioners to optimize model performance for a range of practical uses. The confusion matrix provides a detailed investigation of the model’s predictions regarding ground truth labels. Its four components are false positives (FP), false negatives (FN), true positives (TP), and true negatives (TN). These components can yield a number of performance metrics, including the F1 score, recall, and precision through the Equations (6), (7), and (8), respectively. These metrics offer an in-depth assessment of the model’s accuracy, highlighting its accuracy in recognizing instances that are both positive and negative. The accuracy calculation is shown in Equation (9):

(6) $$F1\;\mathrm{Score}=2\times \frac{\mathrm{Precision}\times \mathrm{Recall}}{\mathrm{Precision}+\mathrm{Recall}}$$

(7) $$\mathrm{Recall}=\frac{\mathrm{TP}}{\mathrm{TP}+\mathrm{FN}}$$

(8) $$\mathrm{Precision}=\frac{\mathrm{TP}}{\mathrm{TP}+\mathrm{FP}}$$

(9) $$\mathrm{Accuracy}=\frac{\mathrm{TP}+\mathrm{TN}}{\mathrm{TP}+\mathrm{TN}+\mathrm{FP}+\mathrm{FN}}$$

**Figure 4:**
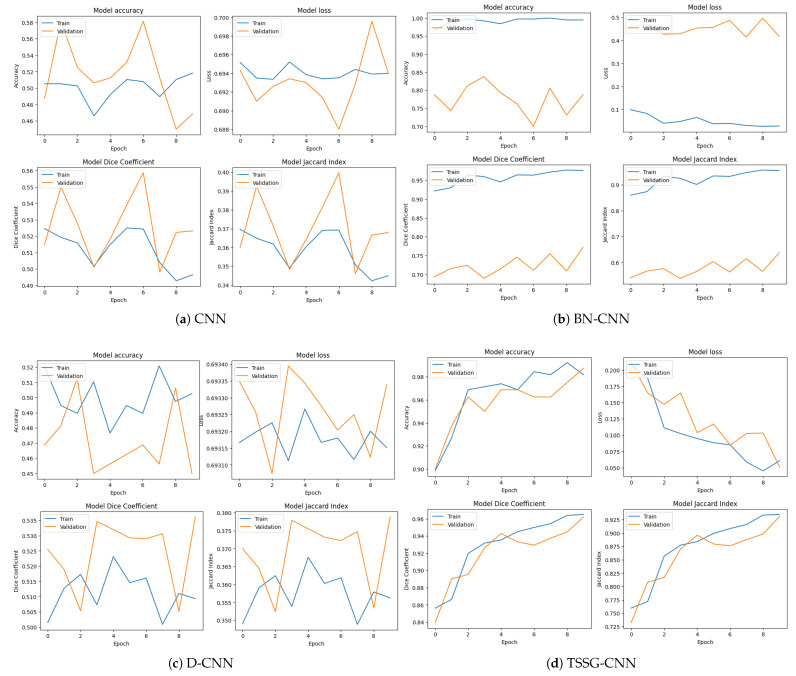
Accuracy, loss, Dice coefficient, and Jaccard index curves for training and testing data.

Loss curves, accuracy curves, Jaccard index curves, and Dice coefficient curves are presented in Figure 4 for all the models utilized. Loss curves show how the model’s loss function—such as cross-entropy loss—evolved throughout training epochs and provide insights into the convergence and optimization process. The model’s accuracy curves show the variations between the training and validation accuracy curves, which helps indicate issues with the complexity of the model or dataset biases. The Dice coefficient is also termed Dice’s Similarity Coefficient, symbolized as DSC, and is a statistical measure used to compare a rate between sets. It is calculated as the extent of agreement of the polarity between the segmentation and the ground truth in terms of area, estimated as twice the surface area of the intersection of the predicted segmentation with the ground truth, normalized with respect to the total number of pixels in both the predicted as well as the ground truth segmentations. The size of the intersection set divided by the size of the union set measures the Jaccard index or the Intersection over Union (IoU). It is measured as the area of overlap between the predicted segmentation and the reference segmentation divided by the area of the union of these two segments.

Every model’s loss curve, accuracy curves, Jaccard index curves, and Dice coefficient curves are shown as a whole, e.g., Figure 4d presents all the four metrics curves that were calculated during training of the TSSG-CNN model. The metrics show highly accurate results for prediction by providing accurate graphs. On the other hand, DCNNs in Figure 4c show irregularity in the curves with low accuracy. The Jaccard index and Dice coefficient curves show low curves.

Table 2 provides an overview of the metrics employed for the accuracy, precision, recall, and F1-score evaluations of the TSSG-CNN model. Specifically, the TSSG-CNN model achieved an exceptional training accuracy score of 98.75%, outperforming previous approaches in the field. Following training, a thorough evaluation of the model’s robustness and generalization capacity was conducted using previously encountered images. Even with these new images offering accurate predictions for TB, the TSSG-CNN model surprisingly demonstrated amazing predictive accuracy. This illustrates how well the model generalizes to new data, which is crucial in real-world scenarios where seeing unusual events is frequent.

**Figure 5:**
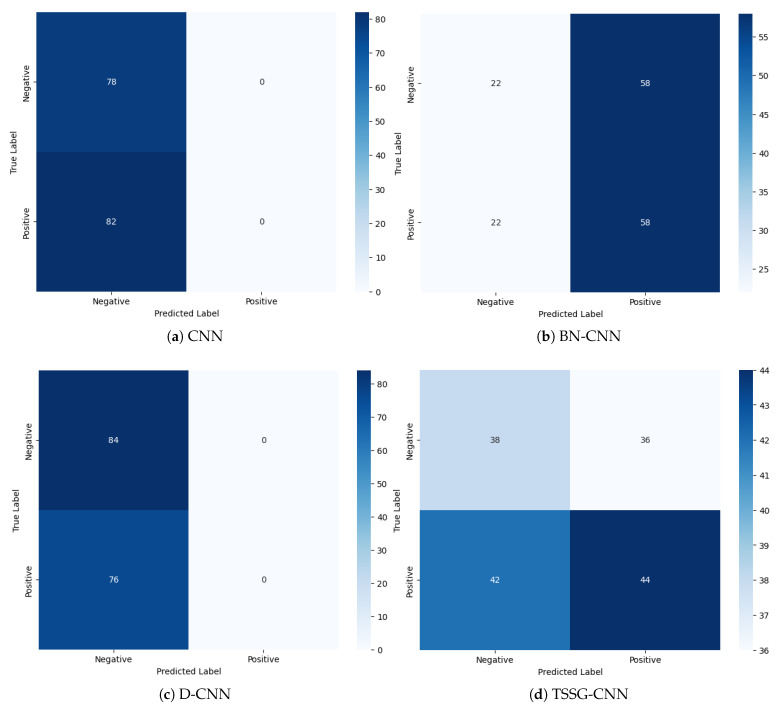
Confusion matrix of the proposed models.

The remarkable accuracy of the TSSG-CNN model in training and testing indicates how well it addresses the underlying issue and increases the likelihood that it will be helpful in practical settings. These results validate the effectiveness of the TSSG-CNN model and emphasize its significance in pushing the boundaries of the current state-of-the-art in the area. The classification model performs admirably across two classes, as seen by the confusion matrix in Figure 5d. The model correctly classifies 38 examples as falling into the first class and 41 instances as belonging to the second class, demonstrating its ability to distinguish between the two classes. The relatively low number of class misclassifications by the model serves as additional evidence of its efficacy. On the other hand, CNN and D-CNN models in Figure 5a,c were not able to differentiate between the two classes and generated false results; meanwhile, BN-CNN in Figure 5b was able to differentiate between them, but its results were not as accurate, since it misclassified instances.

**Figure 6:**
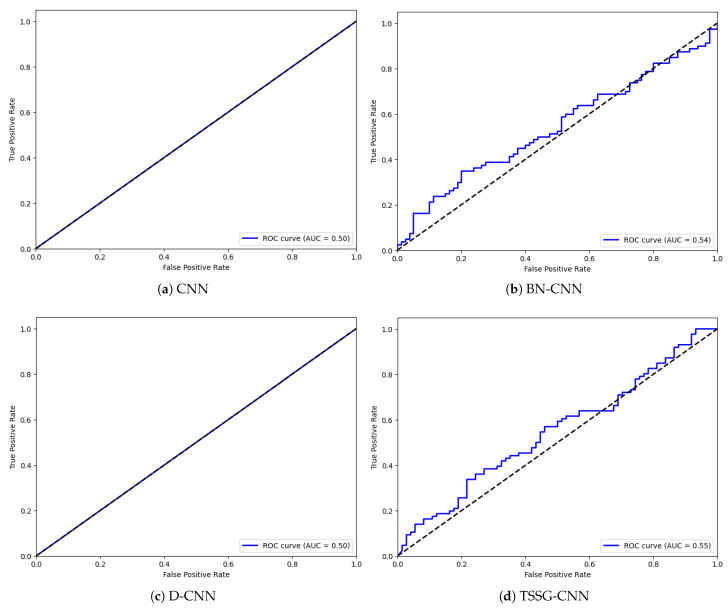
ROC curves of the proposed models.

The same was performed with Receiver Operating Characteristic (ROC) curves, just as confusion matrices were used to examine the poor-performing classes out of the target column. The Receiver Operating Characteristic (ROC) curves are presented in Figure 6 to draw the true positive rates against false positive rates. The model TSSG-CNN represented in Figure 6d developed curves of good model performance in the new environment. At the same time, BN-CNN represented in Figure 6b also presented good outcomes. However, Figure 6a,c of both the CNN and the D-CNN have plotted relatively less accurate curves, which signify the poor performance of these models.

The evaluation results of different deep learning models are presented in Table 3, along with the performance metrics used for evaluation consisting of accuracy, F1-score, precision, and recall. Four models were evaluated on this dataset: a basic convolutional neural network model, a batch normalization CNN model, a multi-modal CNN and Dense Layer model, and the TGSD that consists of a segmentation and a classification model; these models achieved accuracies of 50.63%, 79.38%, 42.5%, and 98.75% respectively.

**Figure 7:**
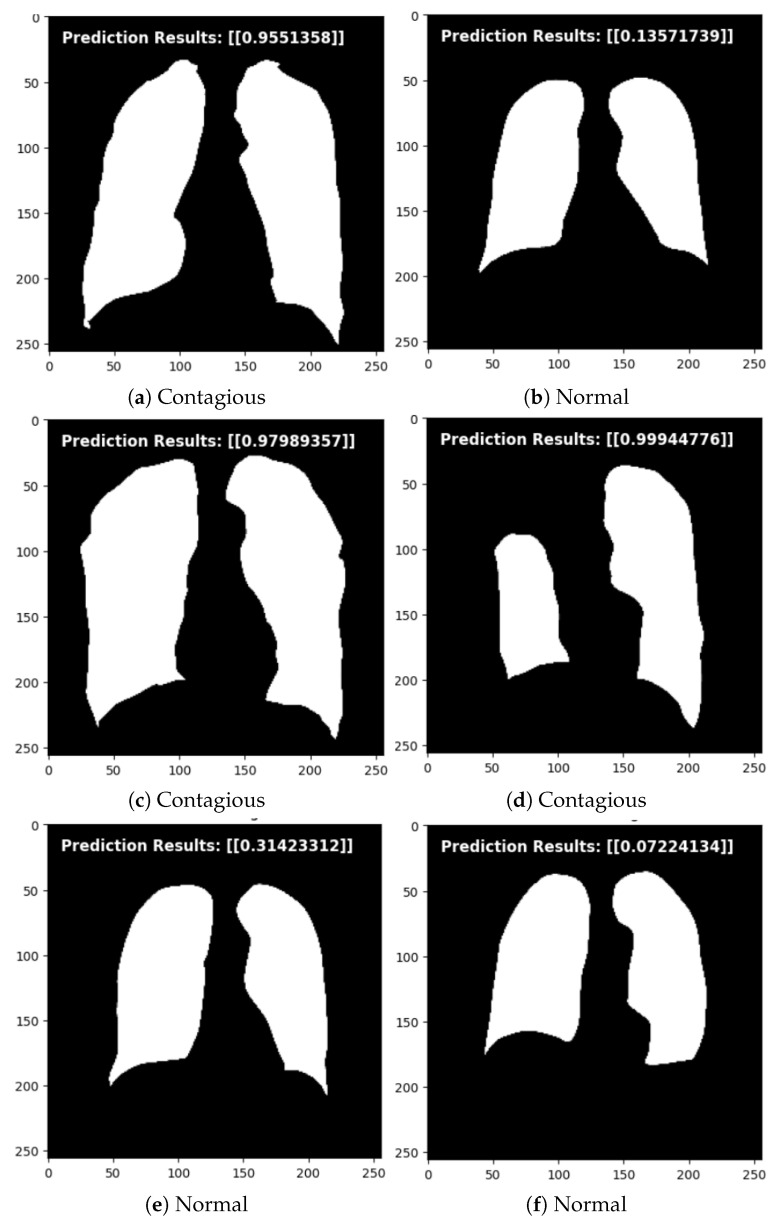
Prediction results on both classes.

The evaluation of these models showed that the TSSG-CNN and BN-CNN models performed well, while the other two models, CNN and DCNN, produced poor results. Furthermore, in the testing phase, the newly proposed model TSSG-CNN performed better than BN-CNN, which is evident in Figure 7.

For TSSG-CNN robustness, we experimented with various segmentation and classification model sizes, as shown in Table 4. To determine the optimal architecture for the hybrid model, an ablation study was conducted by testing four different configurations: some of the techniques involved are small, medium, and large. The image set included 566 images, and the distribution to training, validation, and testing sets was eight-zero-one, six-zero-one and six-five-six, respectively. In the smallest model, the layers and the filters were comparatively less, and in the second model, there were a moderate number of layers and filters, while in the third largest model, the layers and the filter were the maxima. These findings further indicate that as the model complexity increases, the likelihood of a correct classification increases as well, meaning that the architectural design of a deep learning model is crucial in improving the efficiency of medical imaging solutions.

**Figure 8:**
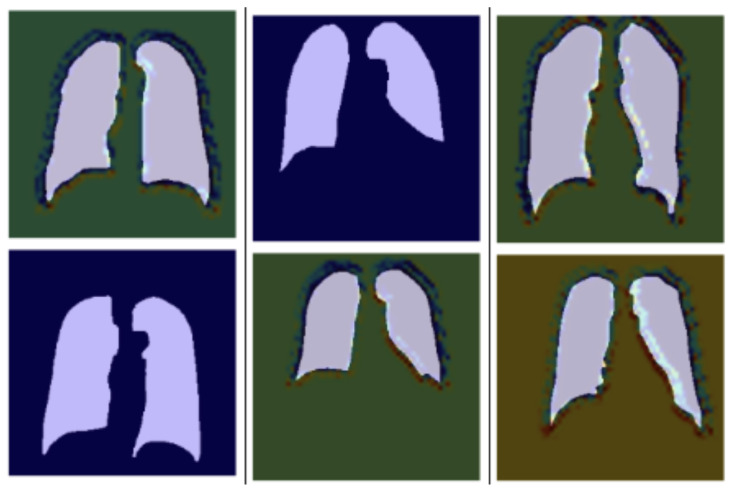
Grad-cam visual representation.

For a better understanding of health professionals about the dataset and its results, the Grad-cam visualization technique was used as shown in Figure 8. Grad-CAM (Gradient-weighted Class Activation Mapping) is an innovative strategy that helps increase the interpretability of deep learning approaches, primarily in the area of image classification and segmentation. Destined to become increasingly popular in the course of deep learning, Grad-CAM can help to understand which areas of an input image the model pays the most attention to. This technique quantifies the feature maps of the final convolutional layer by computing gradients of the predicted class with regard to these maps. Grad-CAM then places these importance scores on top of the original image, allowing it to produce heatmaps that show where the various components attend. Grad-CAM helps explain the proceedings of an intricate neural network as it generates outcomes that are easier for other users to comprehend and, therefore, relies more on the model’s credibility. Such interpretability is especially important in the medical field, where knowledge and transparency of the AI output are crucial for doctors to understand what an AI model is thinking and to come up with the best diagnosis/treatment plans.

### Discussion and Comparison

Four distinct deep-learning models were evaluated using an image dataset of 558 images of X-rays of lungs collected for Shenzen Hospital. The evaluation results for the TSSG-CNN Model are presented in Table 2 and Table 3, presenting the results of all the models; the TSSG-CNN Model provided an accuracy of 98.75%, surpassing the baseline approach with a margin of 4.3%. The evaluation was based on accuracy, precision, recall, and F1-score. Table 5 represents the comparison between the proposed approach of this paper and the baseline approach.

The TB Segmentation-Guided Detection (TSSG-CNN) model was tested using a set of never-before-seen images after being trained on a dataset comprising images of both contagious and normal cases of TB. To assess the model’s capacity for generalization and precise TB case classification, a set of carefully chosen images representing a range of situations and settings was fed into the model. The TSSG-CNN model performed remarkably well, efficiently differentiating between typical cases and those that suggested TB transmission, as seen in Figure 7. The model demonstrated its effectiveness in real-world circumstances outside of the training dataset by effectively utilizing learned features to identify important patterns and traits indicative of TB infection through the use of deep learning architecture.

The application of the TSSG-CNN model for clinical use has to address a number of different aspects to enable integration and efficiency. In the world of computing, high-end GPUs like NVIDIA Tesla or A100, fast multi-core CPUs of the Intel Xeon family, at least 16 GB of RAM and enough SSD space are required. The software stack should contain TensorFlow/Keras (specifically preferred for Linux for better GPU support) and all the required libraries and dependencies. PACS should comply with existing healthcare systems’ data sharing standards—HL7 and DICOM, data encryption requirements and regulations such as GDPR and HIPAA. A graphical user interface through which the machine segmentation process can be linked with the electronic health records (EHR) and visualization of segmentation is also necessary. The use of real clinical information, support for decisions on a larger scale, and training/implementation assistance for clinical personnel are also crucial. Regarding ethical and legal issues, algorithmic transparency addressing biases will have to be addressed so as to deliver the levels of reliability and validity needed in clinical practice.

## 5. Conclusions and Future Scope

This paper suggested a deep-learning CNN model for the detection of TB through X-ray images. The proposed approach, named TSSG-CNN, consists of a segmentation model followed by a classification model for prediction. The extensive evaluation provided informative data regarding the TB Detection system’s reliability and usefulness. It gave us better results than the previous approaches, with an increase of 5.55% in accuracy and 1.5% in F1-score, providing this paper with 98.75% accuracy and 98.70% F1-score. It also provided higher Jaccard index and Dice coefficient values of 95.33% and 98.75%, respectively. When tested on unseen images, the model’s performance was confirmed to be durable, and it kept proving to be incredibly accurate and efficient. These outcomes highlight the model’s ability to generalize over a wide variety of data. The evaluation results imply that the proposed approach can generate better results on the available dataset. Additionally, we plan to investigate the incorporation of multimodal data sources, including genetic data or clinical metadata, which may help develop more sophisticated diagnostic tools and provide a more thorough understanding of TB detection.

## Figures and Tables

**Table 1:** Summary of the related work.

Paper	Key Findings
[12]	- Proposed the “Incremental Learning-based Cascaded Model” (ILCM) for TB identification in chest X-ray (CXR) photos. - Automated identification of infected areas and classification of TB cases. - Achieved an F1 score of 97.23% and overall accuracy of 93.20% on local data.
[13]	- Combined explainable Artificial Intelligence (XAI) techniques with CNN framework for TB detection in chest X-rays. - Accuracy between 98.7% and 99.1%. - Improved interpretability of CNN’s decision-making process.
[14]	- Utilized the Mayfly Algorithm (MA) and Dual Deep Learning Features for TB detection in CXR images. - Achieved accuracy rates of 97.8% using the KNN classifier. - Optimized feature selection and refinement using MA.
[15]	- Proposed an IoT-based healthcare application for early TB diagnosis using CXR images. - Used Adaptive Fuzzy C-means clustering and Deep Belief Network (DBN) for feature extraction and classification. - Performance improvement using Adaptive Monarch Butterfly Optimization (AMBO) method.
[16]	- Introduced a pipeline for automated TB screening in chest X-rays using deep learning. - Combined three deep learning architectures and applied various techniques for improved performance. - Achieved 97.1% classification accuracy and high evaluation metrics scores.
[17]	- Developed CBAMWDnet model for early TB identification using CXR images. - Combined Convolutional Block Attention Module (CBAM) and Wide DenseNet (WDnet) for improved feature extraction. - Achieved 98.80% accuracy and performed well on evaluation metrics.
[18]	- Proposed a deep learning model for joint diagnosis of lung disorders using chest X-rays. - Trained on publicly available Kaggle datasets and achieved 98.72% accuracy. - Outperformed existing methods in precision and diagnosis of specific disorders.
[19]	- Introduced a computer-aided diagnostic (CAD) method for automated chest X-ray-based TB identification. - Combined Gabor filters and deep features from pre-trained models for comprehensive detection. - Achieved high area under the curve (AUC) values on evaluation datasets.
[20]	- Addressed class disparity in the TBX11K dataset using the Synthetic Minority Over-sampling Technique (SMOTE). - Evaluated performance of Random Forest (RF) and XGBoost (XGB) models with and without SMOTE. - SMOTE improved the precision–recall trade-off, but slightly decreased overall accuracy.
[21]	- Explored deep learning models for chest X-ray quality control (QC) in TB testing. - Demonstrated exceptional performance in identifying anomalous X-rays and anomalies related to TB. - Good performance on external datasets.

**Table 2:** Evaluation results on validation data.

Model	TSSG-CNN
Accuracy	98.75%
F1-Score	98.70%
Precision	97.43%
Recall	100%
Dice coefficient	98.75%
Jaccard index	95.33%

**Table 3:** Evaluation results.

Model	CNN	BN-CNN	DCNN	TSSG-CNN
Accuracy	50.63%	79.38%	42.5%	98.75%
F1-score	67.22%	74.81%	59.65%	98.70%
Precision	50.63%	100%	42.5%	97.44%
Dice coefficient	49.73%	97.37%	52.29%	98.75%
Jaccard index	34.55%	65.29%	36.69%	95.33%

**Table 4:** TSSG-CNN implemented models.

Model	Accuracy
Small	50.00%
Medium	85.71%
Large	57.14%
Originally used	98.75%

**Table 5:** Performance comparison with [12].

Reference	Accuracy
[12]	93.20%
TSSG-CNN	98.75%

## Data Availability

The dataset is publicly available.
